# The Convergent Evolution of Blue Iris Pigmentation in Primates Took Distinct Molecular Paths

**DOI:** 10.1002/ajpa.22280

**Published:** 2013-05-02

**Authors:** Wynn K Meyer, Sidi Zhang, Sachiko Hayakawa, Hiroo Imai, Molly Przeworski

**Affiliations:** 1 Department of Human Genetics, University of ChicagoChicago, IL 60637; 2 Biological Sciences Collegiate Division, University of ChicagoChicago, IL 60637; 3 Department of Behavioral and Brain Sciences, Cognition and Learning Section, Primate Research Institute, Kyoto UniversityInuyama, Aichi 484-8506, Japan; 4 Department of Cellular and Molecular Biology, Molecular Biology Section, Primate Research Institute, Kyoto UniversityInuyama, Aichi 484-8506, Japan; 5 Department of Ecology and Evolutionary Biology, University of ChicagoChicago, IL 60637; 6 Howard Hughes Medical Institute, University of ChicagoChicago, IL 60637

**Keywords:** parallel evolution, adaptation, predictability, pleiotropy

## Abstract

How many distinct molecular paths lead to the same phenotype? One approach to this question has been to examine the genetic basis of convergent traits, which likely evolved repeatedly under a shared selective pressure. We investigated the convergent phenotype of blue iris pigmentation, which has arisen independently in four primate lineages: humans, blue-eyed black lemurs, Japanese macaques, and spider monkeys. Characterizing the phenotype across these species, we found that the variation within the blue-eyed subsets of each species occupies strongly overlapping regions of CIE L*a*b* color space. Yet whereas Japanese macaques and humans display continuous variation, the phenotypes of blue-eyed black lemurs and their sister species (whose irises are brown) occupy more clustered subspaces. Variation in an enhancer of *OCA2* is primarily responsible for the phenotypic difference between humans with blue and brown irises. In the orthologous region, we found no variant that distinguishes the two lemur species or associates with quantitative phenotypic variation in Japanese macaques. Given the high similarity between the blue iris phenotypes in these species and that in humans, this finding implies that evolution has used different molecular paths to reach the same end. Am J Phys Anthropol 151:398–407, 2013.© 2013 Wiley Periodicals, Inc.

In natural populations, the number of possible genetic changes leading to a given trait is determined by the combination of two factors: the number of loci at which mutations influence the phenotype (the mutational target size) and the proportion of these mutations that do not have prohibitively deleterious consequences on other phenotypes (the extent of pleiotropy; Stern and Orgogozo, 2008; Stern, 2011). Examples of convergent evolution, the independent acquisition of the same trait in different populations or species, may be highly informative with regard to questions of evolutionary predictability and constraint because these likely represent multiple solutions to similar selective pressures (see, e.g., Conway Morris, 2003; Christin et al., 2010; Losos, 2011).

To date, cases of convergence have been found to occur through independent coding changes in the same gene (e.g., Mundy, 2005; Christin et al., 2008; Gross et al., 2009; Kingsley et al., 2009); through changes in distinct non-coding elements regulating the same gene (e.g., Miller et al., 2007); through distinct changes in the same regulatory element (e.g., Tishkoff et al., 2007); or even through changes in the same protein domains (e.g., Aminetzach et al., 2009; Feldman et al., 2012) or at the same amino acids (e.g., Li et al., 2010; Liu et al., 2010b; Shen et al., 2012). In other cases, changes in distinct loci result in the evolution of the same trait (e.g., Chen et al., 1997; Hoekstra et al., 2006; Borowsky, 2008; Steiner et al., 2009). Most studies have found that convergent phenotypes tend to evolve through changes within the same gene (Conte et al., 2012; Martin and Orgogozo, in press) or within a particular pathway (e.g., Chan et al., 2012; Tenaillon et al., 2012); in the absence of a reporting bias, these findings indicate that the number of permissible paths to a trait is often limited.

Many of these reported cases of phenotypic convergence involve pigmentation (see Hubbard et al., 2010), notably in the color of skin (Miller et al., 2007; Gross et al., 2009), fur (Hoekstra et al., 2006; Kingsley et al., 2009; Steiner et al., 2009), or plumage (Mundy, 2005). In primates, blue iris pigmentation has been documented in four different lineages. In humans (*Homo sapiens*), the prevalence of the trait increases with latitude from 24 to 55% in European populations (Zanetti et al., 1996; Laeng et al., 2007); it has also been observed in populations with European admixture (Frudakis et al., 2007). In Japanese macaques (*Macaca fuscata*), which are endemic to the broad-leaved deciduous and evergreen forests of Japan (Oi, 2002; Abe et al., 2005), blue or intermediate iris color has been observed at frequencies of 12 to 19% in colonies on Shodoshima and Kyushu islands (Yamagiwa, 1979; Zhang and Watanabe, 2007). Blue irises have also been reported in the brown spider monkey (*Ateles hybridus*, formerly *Ateles belzebuth hybridus*; Hernandez-Camacho and Cooper, 1976; Konstant et al., 1985) and closely related Colombian black spider monkey (*Ateles geoffroyi* or *fusciceps*, subspecies *rufiventris*; Hernandez-Camacho and Cooper, 1976; Defler et al., 2004), which inhabit evergreen, semi-deciduous, and montane seasonal forests in Colombia, Panama, and Venezuela (Mondolfi and Eisenberg, 1979; Defler et al., 2004). Although the prevalence of this trait in these species has not been extensively documented, approximately 13% of a population of brown spider monkeys in north central Colombia has blue irises (R. Rimbach and A. Link, personal communication). In contrast to these other species, all blue-eyed black lemurs (*Eulemur flavifrons*, formerly *Eulemur macaco flavifrons*) have blue irises, whereas in the most closely related species, the black lemur (*Eulemur macaco*, formerly *Eulemur macaco macaco*), all individuals have brown irises (Mittermeier et al., 2006). These sister species inhabit primary and secondary tropical sub-humid forests in a narrow range in northwestern Madagascar (Rabarivola et al., 1991; Andrianjakarivelo, 2004; Randriatahina and Rabarivola, 2004) and hybridize across part of this range (Rabarivola et al., 1991). Given that almost all other primates have brown or yellow irises (Kobayashi and Kohshima, 2001), blue iris pigmentation can be inferred to be a derived trait that has arisen independently on these lineages.

Beyond these broad strokes, the extent of iris pigmentation variation within and between species has not been characterized. In particular, it is not yet clear whether the reported blue irises represent the same phenotype in all species. Demonstrating such similarity is important, as if the derived phenotypes were different, we would anticipate the involvement of distinct genetic loci. Thus, mapping the traits would not provide additional information with regard to evolutionary constraint. Quantitative measures of iris pigmentation variation, recently demonstrated to be associated with genetic variation in humans (Liu et al., 2010a; Edwards et al., 2012), provide a way of evaluating phenotypic similarity across species.

In humans, the phenotype of blue iris pigmentation has well understood molecular and genetic underpinnings. Observable human “eye color” depends upon the amount and type of melanin within the outermost layers of the iris (Prota et al., 1998; Wielgus and Sarna, 2005). Whether human irises are blue or brown can be predicted with >80% accuracy by the genotype at a single nucleotide polymorphism (SNP), rs12913832, in a regulatory element influencing the expression of *OCA2* (Eiberg et al., 2008; Sturm et al., 2008; Liu et al., 2009; Visser et al., 2012). The *OCA2* gene itself encodes a transport protein expressed in the iris that influences the development of melanocytes (Rinchik et al., 1993; Brilliant, 2001). In addition to the main effect SNP, variants within *OCA2* and at least 12 other genes have been more weakly associated with human iris color variation; these likely act as modifiers of iris color with smaller effect size (Kanetsky et al., 2002; Frudakis et al., 2003; Graf et al., 2005; Duffy et al., 2007; Kayser et al., 2008; Sturm et al., 2008; Liu et al., 2009). Age strongly influences human iris color variation, and sex has a significant but weak effect (Liu et al., 2010a).

Our understanding of the genetic basis of blue iris pigmentation in humans can inform hypotheses regarding the genetic basis in other primates. In humans, several coding sequence mutations and deletions of coding regions in *OCA2* cause oculocutaneous albinism, or severely reduced pigmentation of the hair, skin, and irises (Manga and Orlow, 1999; Oetting and King, 1999), whereas regulatory SNP rs12913832 has a more moderate influence on hair and skin phenotypes (Sulem et al., 2007; Branicki et al., 2009). If dramatic pigmentation reduction in skin or fur were deleterious in other primates, we may expect blue irises to evolve via regulatory mutations in these species as well. Notably, SNP rs12913832 lies within a stretch of 410 bp demonstrating strong conservation among mammals; thus, this region is a strong candidate for variants with the capacity to influence *OCA2* expression. Bradley et al. (2009) sequenced 166 bp of the orthologous region and found no fixed differences between the two lemur species. They identified one variant that was polymorphic within the blue-eyed black lemurs but not the black lemurs sequenced, a pattern that could be suggestive of this variant or a linked site contributing to pigmentation differences.

Several lines of evidence support the idea that blue irises have fitness effects in humans. The rarity of observation of the phenotype in nature, in combination with observed deleterious pleiotropic consequences in humans (Eagle Jr, 1994; Imesch et al., 1997; Metallinos et al., 1998; Santschi et al., 1998; Yang et al., 1998; Smith et al., 2000; Pingault et al., 2010) and domesticated animals (Juraschko et al., 2003; Geigy et al., 2007; Hauswirth et al., 2012), suggests that some benefit would be required to overcome purifying selection against these pleiotropic effects. Population genetic evidence also supports recent positive selection at the locus responsible for the majority of the phenotypic variation in humans (Voight et al., 2006; Donnelly et al., 2012; Yang et al., 2012). Although the mechanism driving this population genetic signature is unclear, direct selection for blue irises to improve short wavelength perception (Bornstein, 1973; Laeng et al., 2007), selection for light skin pigmentation to increase vitamin D absorption (see, e.g., Jablonski and Chaplin, 2000; Parra, 2007), and sexual selection (Darwin, 1871; Diamond, 1992; Aoki, 2002; Frost, 2006; Laeng et al., 2007) may have played a part.

Evidence for an adaptive value of blue irises in non-human primates is more limited; however, pleiotropy and sexual selection may also have played a role in these species. In Japanese macaques, the presence of blue irises is moderately correlated with reduced pigment in fur (Yamagiwa, 1979), so selection on lighter fur could also have increased the frequency of blue irises. Of note, Japanese macaques have the northern-most range of any non-human primate (30°21' to 41°08'; Oi, 2002), so selection either for increased absorption of UVB or for short wavelength perception could have occurred in this species. In contrast, neither blue-eyed black lemurs (which are more lightly pigmented than black lemurs among females; Mittermeier et al., 2008) nor either species of spider monkey with blue irises live at extreme latitudes. Several adaptive hypotheses involving blue irises, including sexual selection and species recognition, have been proposed for the blue-eyed black lemur (Bradley et al., 2009). Alternatively, blue irises may have risen to high frequency by chance (i.e., via genetic drift) in the non-human primate species. Identifying the loci that underlie blue iris pigmentation in these species would help in evaluating hypotheses about the selective pressures shaping the evolution of this phenotype.

## MATERIALS AND METHODS

### Obtaining and selecting photographs

Photographs of five black lemurs and eleven blue-eyed black lemurs from the Duke Lemur Center (DLC), Durham, North Carolina, were kindly provided by David Haring (DLC). Photographs of 42 Japanese macaques housed at the Primate Research Institute (PRI) in Inuyama, Aichi Prefecture, Japan, were taken by PRI staff. We obtained photographs of 19 free-ranging Japanese macaques in the Choshikei Monkey Park on Shodoshima Island, Kagawa prefecture, Japan. Photographs of eight brown spider monkeys were kindly provided by Rebecca Rimbach at the German Primate Center, Göttingen. Photographs of 119 humans of European ancestry were kindly provided by Esteban Parra and Melissa Edwards of the University of Toronto, Mississauga. Detailed information about photographic methods may be found in Supporting Information Section 1. We removed any photographs that had low resolution, were too dark to recognize iris color by eye after white balance adjustment, or did not include the complete iris, leading us to exclude nine captive Japanese macaques and two brown spider monkeys. For individuals with multiple photographs of sufficient quality, we randomly selected one for analysis.

### Processing and summarizing iris pixels

We applied white balance to each photograph using Adobe® Photoshop® automatic white balance adjustment and selected the iris with Adobe® Photoshop® CS4 quick selection tool. We excluded all pixels with RGB values higher than 250 (black) or lower than 10 (white), which represent shadows cast by the eyelids and eyelashes or reflections of light in the iris. We used the CIE L*a*b* color space, in which L* represents brightness, a* represents relative amount of magenta to green, and b* represents relative amount of yellow to blue, for further analysis (Malacara, 2002). We converted from RGB to CIE L*a*b* using custom code by Mark Ruzon downloaded from http://www.mathworks.com/matlabcentral/fileexchange/24009 on January 25, 2012, and calculated the median of a* and b* (excluding L* because of its dependence upon lighting conditions). Further description of the choice of white balance method, consistency of iris selection, and choice of color system and summary statistic may be found in Supporting Information Sections 2 to 4.

### Test of two clusters versus one

To test a two cluster model of the phenotype distribution against a one cluster model within each primate group, we fitted a mixture normal model with two components and compared the likelihood to that of a simple normal model using a likelihood ratio test (Supporting Information Table S1). Models were fitted using the mixtools version 0.4.4 (Benaglia et al., 2009) and fitdistrplus (Delignette-Muller et al., 2010) packages in R 2.13.1, and an empirical null distribution of the test statistic was generated by calculating test statistics for 100,000 datasets simulated from the simple normal model using the maximum likelihood estimate (MLE) of parameters (see Supporting Information Section 5). In order to minimize the effects of relatedness in the lemurs, we included only minimally related individuals (no relationships closer than avuncular), resulting in a sample size of eight. In order to reproduce the effects of this limited sample size in humans and Japanese macaques, we selected eight individuals by randomly sampling the minimally related individuals, and we performed tests of clustering on 1,000 such random samples.

### DNA samples

Blood or DNA samples were obtained for eight of the photographed Japanese macaques housed at the PRI. DNA was extracted when necessary using the QIAamp DNA mini kit (Qiagen). Fecal samples were collected non-invasively from nine of the photographed Japanese macaques from Choshikei Monkey Park. Initial visual categorization of these macaques suggested that four (adult females) had blue irises, one (adult female) had irises of intermediate color, and four (two adult females and two juvenile males, one of which was the offspring of the adult female with intermediate iris color) had brown irises. Samples were stored in ethanol and transferred to silica gel after 5 to 12 h. DNA was extracted using the QIAamp DNA Stool kit (Qiagen), using the standard protocol without stool tubes. Four samples (892, 893, 902, and 903) were incubated in buffer ASL for 10 min at 70°C using a heat block in step two to increase DNA yield.

Blood was obtained for six black lemurs and six blue-eyed black lemurs from the Duke Lemur Center (DLC) during annual check-ups or before euthanasia. Two black lemurs (Blanche-Niege and Louie) and two blue-eyed black lemurs (Bogart and Lamour) were also included in Bradley et al. (2009). DNA was extracted using the QIAamp DNA mini kit (Qiagen) and whole genome amplified using the GenomiPhi DNA amplification kit (GE Healthcare).

### PCR and sequencing

Primers were designed to amplify subsets of the conserved region orthologous to the region surrounding SNP rs12913832 in humans (Supporting Information Fig. S1 and Table S2), using the available sequences of *Lemur catta* (GenBank AC126425) and *Macaca mulatta* (GenBank NC_007864). Conditions for PCR protocols are indicated in Supporting Information Table S3. PCR products were purified using Exo-SAP. Sequencing reactions were run using BigDye® on an Applied Biosystems 3730XL or 3130 capillary sequencer (see Supporting Information Section 6).

### Alignment

Sequences were aligned using eBioX v1.6b1 (http://www.ebioinformatics.org) with the default parameters of the Kalign algorithm. Alignments were adjusted manually when this created a consensus among all sequences by eliminating gaps. A *M. mulatta* sequence (GenBank NC_007864) was used to scaffold the alignments from Japanese macaque fecal DNA, and *H. sapiens* sequences (GenBank NC_000015.9 and AC_000147.1) were used to determine position relative to rs12913832. Each base pair of homozygous sequence reported for wild Japanese macaques was supported by at least seven sequencing reads to limit the probability of allelic dropout (i.e., the failure of one of two alternate alleles to amplify) to <0.01 (Navidi et al., 1992; Taberlet et al., 1996). Sequences were considered heterozygous when at least one clean chromatogram peak supported only the alternate allele or at least two were heterozygous (as determined by eye). All sequences are available on GenBank (KC693559-KC693587).

### Association tests

We tested for association of CIE a* and b* with age, sex, origin (captive, i.e., from the PRI, or wild, i.e., from Choshikei Monkey Park) and genotype for Japanese macaques. We first tested for association with adjusted age (two times age divided by maximum age), sex, and origin separately, and we included only significant variables from these analyses as covariates in the genotype association test. To account for relatedness among individuals when known, we fitted linear mixed models using GEMMA v0.91 (Zhou and Stephens, 2012). We used the median of CIE a* and CIE b*, quantile-normalized to a standard normal distribution using R software (R Development Core Team, 2011), as phenotypes. The effects of any covariates were modeled as fixed effects, and the effect of relatedness was modeled as a random effect.

In the lemurs, we performed a test of association at all SNPs, combining the individuals sequenced here and the four additional individuals sequenced in Bradley et al. (2009) for the SNP identified in that study. We implemented a test for a categorical variable indicating species by setting the phenotype value to 2 for black lemurs and to 1 for blue-eyed black lemurs in a mixed model analysis, including a random effect of relatedness, in GEMMA. We used the kinship package in R (Atkinson and Therneau, 2012) to calculate kinship matrices for macaques and for lemurs.

We did not perform tests of clustering or association tests in brown spider monkeys due to limited sample size and photograph quality and possible relatedness within the sample. Age, sex, and known relationship information for all non-human primate samples are listed in Supporting Information Table S4.

## RESULTS

### Evaluating quantitative variation in iris color phenotype

Although some subset of all four species appears to have blue irises, it is unclear whether this “blue” trait is the same in all lineages, and hence whether we expect the degree of similarity in its genetic basis to be informative with regard to evolutionary constraint. Thus, we first assessed the similarity in the derived trait among species. Given the success of quantitative methods in describing heritable human iris color variation and in detecting genetic associations (Liu et al., 2010a; Edwards et al., 2012), we assessed quantitative variation across species using the CIE L*a*b* color system.

**Fig. 1:**
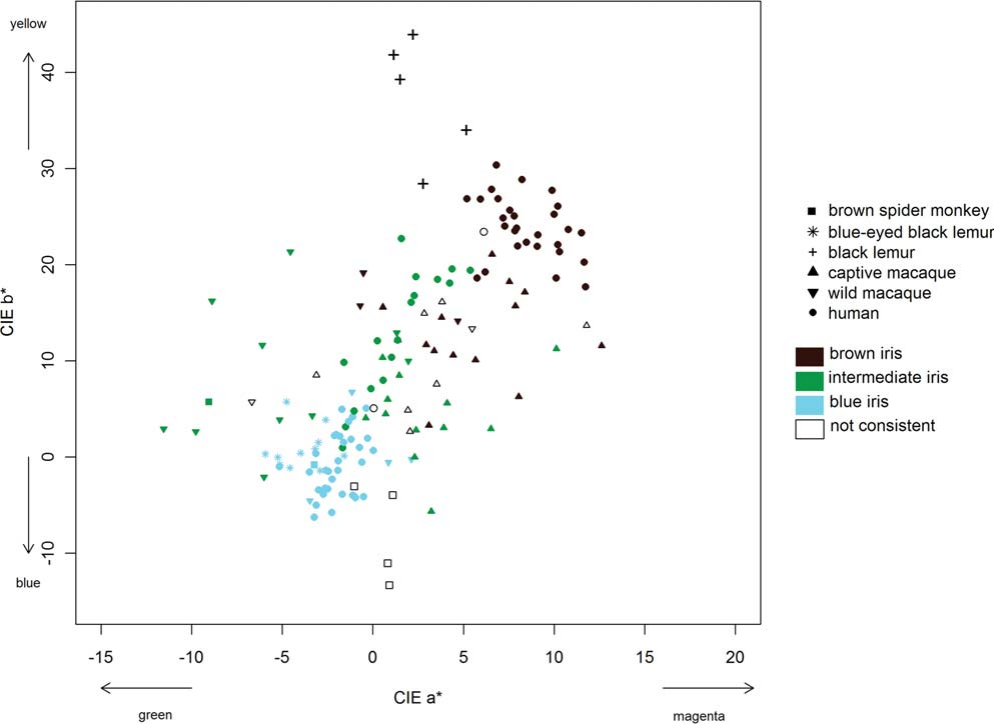
Quantitative phenotypic variation in brown spider monkeys, blue-eyed black lemurs, black lemurs, Japanese macaques and humans; displayed using the CIE L*a*b* color system; with color representing visible color and shape representing species/population. Color of points represents the results of two people (WM and SZ) independently categorizing the irises as “blue,” “brown,” or “intermediate;” “not consistent” indicates that the two people did not agree on the category.

**Fig. 2:**
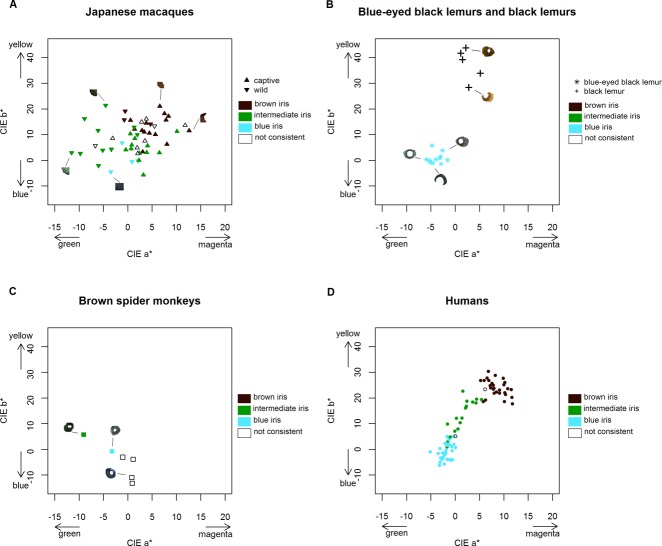
Independent plots for each species or group showing quantitative phenotypic variation in the CIE L*a*b* color system, with color representing visible color (as in Fig. 1). (**A**) Japanese macaques, (**B**) blue-eyed black lemurs and black lemurs, (**C**) brown spider monkeys, and (**D**) humans.

In this color space, individuals with blue irises occupy a highly similar region in all species (Fig. 1). When considering both blue and non-blue irises, however, each species has a slightly different overall phenotypic distribution. Along the magenta-green (CIE a*) axis, lemurs occupy the most restricted region; given that our sample size is limited, a* variation may be greater in natural populations. Despite this limitation, variation on the yellow-blue (CIE b*) axis appears to be greater in lemurs than in any of the other species. Some wild macaques have lower (i.e., more green) CIE a* values than any members of the other species. Considering the relatively complete representation of iris color variation in humans and Japanese macaques in our sample, this may indicate that some macaques have more green irises than humans. Both macaques and humans have continuous variation between blue and brown (Fig. 2A,D), but the relative positions of irises characterized as brown and intermediate differ slightly between species (Fig. 1). Brown spider monkeys occupy the smallest range of any species, which could be due to their limited sample size. Additionally, this species was the least consistently categorized (Figs. 1 and 2C); we suspect that this is a result of the difficulty of assessing color by eye in photographs of animals in the dense forest canopy. The consistently categorized spider monkeys fall within a similar region of the color space to similarly categorized individuals of the other species (Fig. 1).

In the lemurs, iris color can be roughly grouped into two clusters, corresponding to black lemurs and blue-eyed black lemurs (Fig. 2B). In order to test whether a two cluster model fit the phenotypic distributions within each group significantly better than a one cluster model, we performed a likelihood ratio test, comparing the likelihood of a mixture of two normal distributions to that of a simple normal model (see Methods and Supporting Information Section 5). This test indicated a significantly better fit of the mixture model to the black lemur and blue-eyed black lemur CIE b* data in both RAW (a minimally processed image format; *P* = 0.0010) and JPEG (a more processed image format; *P* = 0.00049) data, supporting the presence of two distinct clusters (Supporting Information Table S1). For CIE a*, the mixture model did not fit the data significantly better than the simple normal model in the lemurs (*P* = 0.75 RAW and *P* = 0.11 JPEG). In humans, the mixture model fit the data significantly better than a simple normal model for both color dimensions (a* and b* *P* = 0.000010 JPEG and RAW); the two modes in humans may represent the influence of the main effect SNP. In Japanese macaques, the mixture normal model did not fit significantly better than the simple normal model for CIE a* or b* (*P* = 0.21 and *P* = 0.31, respectively), suggesting that intermediate phenotypes represent a larger proportion of these samples. This may be due to non-random sampling of Japanese macaques with a preference for lighter iris color. When including only eight individuals, as in the lemurs, none of the 1,000 random samples of Japanese macaques and only 3 (JPEG) or 12 (RAW) of the random samples of humans had likelihood ratio test statistics more extreme than the lemurs for CIE b* (Supporting Information Table S4), suggesting that the extreme clustering in lemurs along this axis would not be observed even in a small sample of the other two species.

### Association of environmental variables with quantitative iris color phenotypes

In Japanese macaques, CIE a* values strongly associated with origin (wild vs. captive; *P* = 0.000061). This effect is due to lower (more green) CIE a* values among wild macaques than among captive macaques, a pattern concordant with our observation that more wild macaques possess green or blue iris color than captive macaques. This difference could be due to population structure. In addition, CIE b* values of Japanese macaques were significantly associated with age (*P* = 0.0049): as age increased, CIE b* values decreased (irises become more blue). This result is consistent with previous findings in humans (Liu et al., 2010a). Sex was not significantly associated with quantitative iris color in Japanese macaques, in contrast to the effects found in humans, but this could reflect lack of power.

### Identification and association testing of genetic variants in candidate region

Our sequencing identified seven new variants in blue-eyed black lemurs and one in black lemurs within the 1.2 kb orthologous region surrounding the site of human SNP rs12913832. Neither the newly identified variants nor the haplotypes (in combination with the SNP identified in Bradley et al. (2009)) segregated perfectly with iris color. The previously identified variant, located 1 bp upstream of the human transcription factor binding motif, is polymorphic within blue-eyed black lemurs (at approximately 64% frequency) but fixed for the ancestral allele in the sample of black lemurs sequenced to date (Bradley et al., 2009; Table 1). This interspecific difference was apparent by an association test (see Methods), with *P* = 0.00084 (Table 1). Thus, it is possible that this variant influences iris pigmentation, but if so, another locus or loci must be involved as well, given that the ancestral homozygous genotype at this SNP was observed in one of the six sequenced blue-eyed black lemurs. In contrast, less than 1% of humans with blue irises are homozygous for the ancestral allele at the main effect SNP (Eiberg et al., 2008; Sturm et al., 2008; Branicki et al., 2009).

**TABLE 1:** Variants identified by sequencing the conserved region homologous to the human region surrounding SNP rs12913832

Position^a^	−630	−580	−544	−526	−518	−515	−497	−489	−441	−409	−297	−207	−119	−108	−76	−33	−6	+338
Variant	G > A	G > A	G > A	C > T	C > T	C > T	C > A	G > A	G > T	C > T	A > T	G > A	G > A	C > T	G > A	T > C	C > T	C > T
f_wild_^b^	NA	NA	NA	NA	NA	NA	NA	NA	NA	NA	NA	0	0.056	0.056	0	0	0	NA
f_captive_^c^	NA	0	0	0.13	0.13	0	0.13	0	0	NA	0.19	0.063	0	0	0	0.13	0	NA
f_black_^d^	0	0	0	0	0	0	0	0	0	0	0	0	0	0	0.58	1^e^	0	0
f_blue-eyed_^f^	0.17	0.14	0.14	0	0	0.14	0	0.14	0.14	0.14	0	0	0	0	0	1^e^	0.64	0.33
p_a*_^g^	NA	NA	NA	0.43	0.43	NA	0.28	NA	NA	NA	0.47	0.83	0.44	0.27	NA	0.66	NA	NA
p_b*_^h^	NA	NA	NA	0.94	0.94	NA	0.26	NA	NA	NA	0.40	0.28	0.29	0.41	NA	0.44	NA	NA
p_lemur_^i^	0.27	0.21	0.21	NA	NA	0.21	NA	0.33	0.21	0.47	NA	NA	NA	NA	0.76	NA	0.00084	0.28

a Relative to the base pair homologous to human SNP rs12913832.

b Frequency of the derived allele in the wild Japanese macaque sample.

c Frequency of the derived allele in captive Japanese macaque founders.

d Frequency of the derived allele in black lemur founders.

e The derived state in macaques (inferred from *M. mulatta*) is the ancestral state in lemurs (inferred from *L. catta*).

f Frequency of the derived allele in blue-eyed black lemur founders.

g *P* value of Wald association test with CIE a* in Japanese macaques.

h *P* value of Wald association test with CIE b* in Japanese macaques.

i *P* value of Wald association test with lemur species.

We identified six variants within the 700 bp region sequenced in captive Japanese macaques. None of these variants appeared to predict quantitative iris color (Table 1). Additionally, no variant in the 275 bp sequenced was present in more than one of the nine wild macaques. Thus, it is highly improbable that variation in this region strongly influences iris color in Japanese macaques.

## DISCUSSION

Our quantitative analysis of iris color variation in the four primate species known to have blue irises revealed the blue iris phenotype to be highly similar across species (Fig. 1). Yet our sequencing results indicated that no variant within the region homologous to the causal regulatory region in humans is solely responsible for blue iris pigmentation in either blue-eyed black lemurs or Japanese macaques. Regardless of the genetic architecture underlying the phenotypic variation in these other organisms, the genotype-phenotype map clearly differs from that in humans. Given that the derived phenotype displays marked similarity among species, this implies some level of flexibility in the evolution of this trait.

Our comparison of the phenotypic variation across species suggests hypotheses about the genetic basis of blue irises in Japanese macaques and blue-eyed black lemurs. In humans, iris color varies continuously (e.g., Frudakis et al., 2007; Edwards et al., 2012); however, our perception of this spectrum of variation has led us to categorize iris color into discrete classes: e.g., blue, green, and brown (Kayser et al., 2008; Liu et al., 2010a). The genetic basis of iris color variation in humans reflects both these perceived discrete categories (>80% of the variation in blue vs. brown can be explained by a single mutation) and the underlying continuous nature of the phenotype (several additional loci with more modest effects have been identified; see, e.g., Sulem et al., 2007; Liu et al., 2010a). In Japanese macaques, as in humans, iris color variation is continuous in CIE L*a*b* space (Fig. 2A). This may suggest that one or a few loci have strong effect, producing the perceived blue/brown distinction, with additional genetic or environmental modifiers generating a continuous spectrum between these extremes. In contrast, phenotypic variation is discontinuous between the photographed blue-eyed black lemurs and black lemurs; one or more fixed genetic differences contributing to iris color differences between species could explain this discontinuity. Alternatively, the derived trait may result from a combination of changes in several genes, thus allowing specific variants to segregate within blue-eyed black lemurs. Additional phenotypic data from brown spider monkey populations might enable predictions about the genetic architecture of iris color variation in this species as well.

The observation of a variant just upstream of the human causal variant in the sample of blue-eyed black lemurs, but not the sample of black lemurs, raises the possibility that this variant or one linked to it influences iris pigmentation. Although this variant cannot produce blue irises on its own, it could do so in combination with variation at other loci. Such a complex genetic basis for iris pigmentation could explain the intermediate phenotype observed in hybrids between blue-eyed black lemurs and black lemurs (Meyers et al., 1989; Rabarivola et al., 1991).

The similarity in derived phenotype among humans, Japanese macaques, blue-eyed black lemurs, and brown spider monkeys makes it more likely that causal sites in the non-human primates lie within regions containing variation influencing *OCA2* and other known pigmentation genes in humans. Moreover, given the deleterious effects of many coding mutations in *OCA2* in humans (see http://albinismdb.med.umn.edu) and, more generally, the ability of regulatory variants to produce subtle and tissue-specific changes in phenotype (Stern, 2000; Carroll et al., 2001; Carroll, 2005), regulatory regions may be a priori more likely to contain the causal site or sites than coding regions. These considerations suggest that studies investigating genomic regions involved in human pigmentation in the three non-human primates will enhance our knowledge of the level of similarity in the genetic basis of iris pigmentation across species and inform our understanding of the evolutionary constraint on this convergent phenotype.
