# Supplementary figures and images for: The Convergent Evolution of Blue Iris Pigmentation in Primates Took Distinct Molecular Paths
Source: Am J Phys Anthropol. 2013 May 2;151(3):398–407. doi: 10.1002/ajpa.22280 (PMC3746105; doi:10.1002/ajpa.22280)

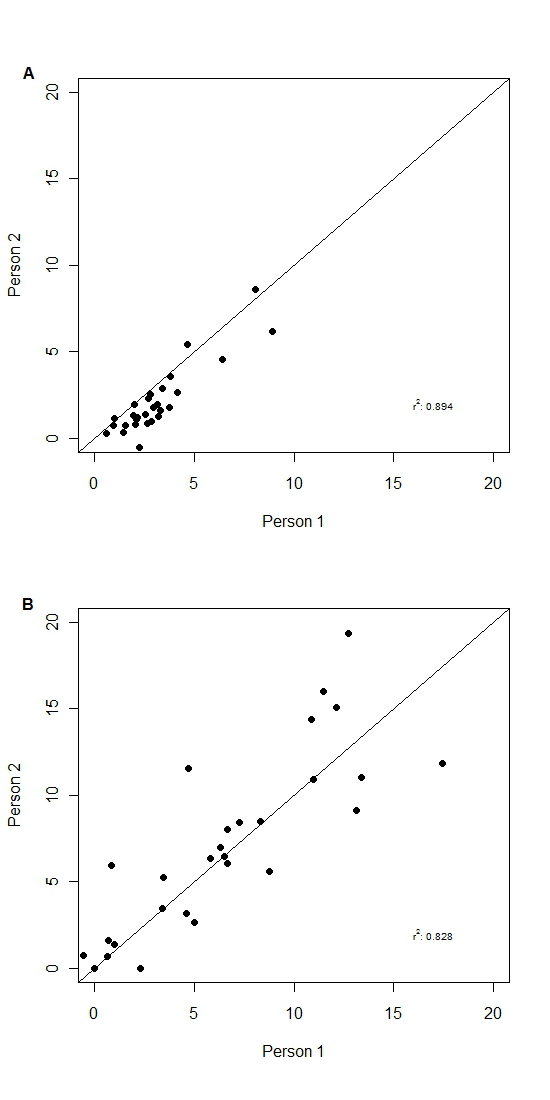

Supplement: Supplementary file 2 [file ajpa0151-0398-SD2.tif]

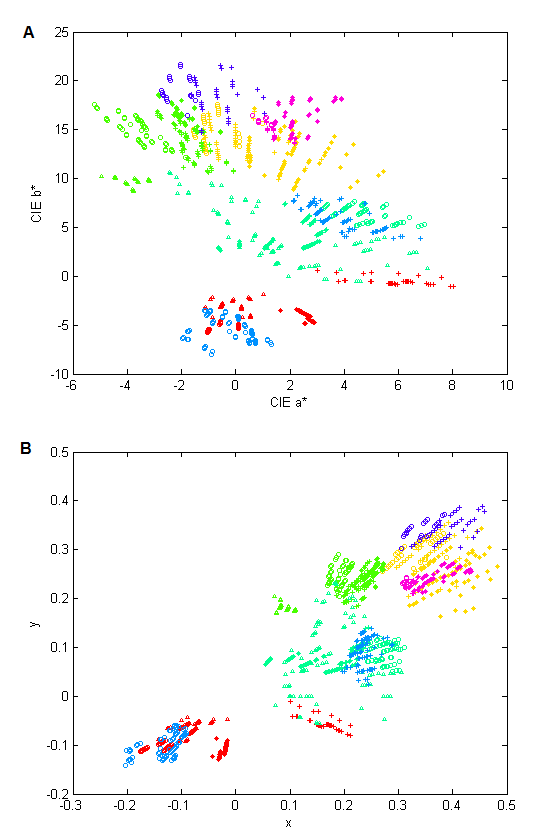

Supplement: Supplementary file 3 [file ajpa0151-0398-SD3.tif]
