# Supplementary material for: The Convergent Evolution of Blue Iris Pigmentation in Primates Took Distinct Molecular Paths
Source: Am J Phys Anthropol. 2013 May 2;151(3):398–407. doi: 10.1002/ajpa.22280 (PMC3746105; doi:10.1002/ajpa.22280)
Supplement: Supplementary file 4 [file ajpa0151-0398-SD4.doc]

**Supporting Information Table S1.** Primer sequences

| **Primer name** | **Sequence** | **PCR protocol** | **PCR/Sequencing** |
| --- | --- | --- | --- |
| Em-116-F | ACACTGGGCTGAAGGTGTTT | 1 | Sequencing |
| L-765-F | CTAACCTGGGCCTCACTGAT | 1 | Sequencing |
| L-687-F | CATGCAGTTCTGGGGCAGGT | 1 | Sequencing |
| Em-1314-R | AGGATTCTGTGGGTCGTTTG | 1 | Sequencing |
| Em-49-F | GCAGCACGATACCCTGAACT | 1 | PCR |
| Em-1462-R | TCAGGCATATTTCCACCACA | 1 | PCR |
| HERC2Spec1-F | GGACATGACTGACGTACAGTTTAGCA | 2 | PCR and Sequencing |
| HERC2Spec3-R | CACATGATTTTTACCCTGATGTTGATT | 2 | PCR and Sequencing |
| HERC2Spec4-F | TGCCCCCAAATATAACTCTGAAACA | 2 | PCR and Sequencing |
| HERC2Spec6-R | AGCCTCAGCCCCTGATGATGATA | 2 | PCR and Sequencing |
| Mf-HERC2cons1a-332u-F | GGTGCCTAACCTGCATCCTA | 3 | PCR and Sequencing |
| Mf-HERC2cons1a-180d-R | GCTCCCACTATCCTCACACC | 3 | PCR and Sequencing |

**Supporting Information Table S2.** PCR conditions

|  | **Protocol 1** | **Protocol 2** | **Protocol 3*** |
| --- | --- | --- | --- |
| total volume | 25 µL | 25 µL | 20 µL |
| DNA | 40 ng | 18 – 457 pg | 30 ng |
| PCR buffer | 1X | 1X | 1X |
| MgCl2 | 1.5 mM | NA | 2.5 mM |
| MgSO4 | NA | 1.25 mM | NA |
| dNTPs | 200 µM | 500 µM | 500 µM |
| each primer | 100 nM | 500 nM | 250 nM |
| Betaine | NA | 1.25 µL | 1 µL |
| DMSO | NA | 1.25 µL | 1 µL |
| Taq | 1 unit (QIAGEN) | 0.5 units Platinum® High Fidelity (Invitrogen) | 1 unit (Invitrogen) |
| initial denaturation | 94ºC for 10 min | 95ºC for 10 min | 94ºC for 5 min |
| denaturation | 94ºC for 30 s | 95ºC for 30 s | 94ºC for 30 s |
| extension | 60ºC for 45 s | 53.5ºC for 30 s | 60ºC for 30 s |
| annealing | 72ºC for 1 min | 72ºC for 30 s | 72ºC for 1 min 30 s |
| final extension | 72ºC for 7 min | 72ºC for 5 min | 72ºC for 10 min |
| cycles | 40 | 40 | 30 |
| samples | lemurs | wild macaques | captive macaques |

*Three PCR products were combined as template for the sequencing reaction.

**Supporting Information Table S3.** Sample information

| **Species** | **Name/ID** | **Age** | **Sex** | **Genotyped?** | **Photograph included?** | **Related individuals** |
| --- | --- | --- | --- | --- | --- | --- |
| *A. hybridus* | AHM1 | 16* | M | N | Y |  |
| *A. hybridus* | AHF1 | 15* | F | N | Y |  |
| *A. hybridus* | AHF2 | 18* | F | N | Y |  |
| *A. hybridus* | Bachue | 10.5* | F | N | Y |  |
| *A. hybridus* | Pepa | 10.5* | F | N | Y |  |
| *A. hybridus* | Vitor | 8.5* | M | N | Y |  |
| *E. flavifrons* | Bogart | 15 | M | Y | N | Barrymore (offspring), Olivier (offspring), Quinn (offspring) |
| *E. flavifrons* | Barrymore | 14 | M | Y | N | Bogart(father), Olivier(brother), Harlow (offspring), Quinn (half brother) |
| *E. flavifrons* | Lamour | 14 | F | Y | Y | Kidman (offspring), Tarantino (offspring) |
| *E. flavifrons* | Redford | 10 | M | Y | Y | Hopkins(brother) |
| *E. flavifrons* | Harlow | 14 | M | Y | Y | Barrymore(father), Quinn (half brother) |
| *E. flavifrons* | Kidman | 12 | F | Y | N | Lamour(mother), Tarantino (half brother) |
| *E. flavifrons* | Hopkins | 15 | M | N | Y | Redford(brother) |
| *E. flavifrons* | Margret | 1 | F | N | Y | Olivier(father), Presley(brother), Aykroyd (brother), Belushi (brother) |
| *E. flavifrons* | Presley | 1 | M | N | Y | Olivier(father), Margret(sister), Aykroyd (brother), Belushi (brother) |
| *E. flavifrons* | Tarantino | 12 | M | N | Y | Lamour(mother), Kidman (half sister) |
| *E. flavifrons* | Olivier | 20 | M | N | Y | Bogart(father), Barrymore(brother), Quinn (half brother), Belushi (offspring), Aykroyd (offspring), Presley (offspring), Margret (offspring) |
| *E. flavifrons* | Quinn | 10 | M | N | Y | Bogart(father), Barrymore (half brother), Olivier (half brother), Harlow (half sister) |
| *E. flavifrons* | Aykroyd | 2 | M | N | Y | Olivier(father), Belushi(brother), Presley (brother), Margret (sister) |
| *E. flavifrons* | Belushi | 2 | M | N | Y | Olivier(father), Aykroyd(brother), Presley (brother), Margret (sister) |
| *E. macaco* | Hesperus | 25 | M | Y | Y |  |
| *E. macaco* | Harmonia | 25 | F | Y | Y |  |
| *E. macaco* | Blanche-Niege | 15 | F | Y | N |  |
| *E. macaco* | Louie | 18 | M | Y | N | Latona (half sister) |
| *E. macaco* | Deucalion | 18 | M | Y | Y | Epimetheus (half brother), Teucer (half brother) |
| *E. macaco* | Epimetheus | 11 | M | Y | Y | Latona(mother), Teucer (brother), Deucalion (half brother) |
| *E. macaco* | Teucer | 22 | M | N | Y | Latona(mother), Epimetheus (brother), Deucalion (half brother) |
| *E. macaco* | Latona | 27 | F | N | Y | Epimetheus (offspring), Teucer (offspring), Louie (half brother) |
| *M. fuscata* (captive) | 937 | 24 | F | N | Y |  |
| *M. fuscata* (captive) | 1006 | 23 | F | N | Y |  |
| *M. fuscata* (captive) | 1102 | 22 | F | Y | Y |  |
| *M. fuscata* (captive) | 1304 | 19 | M | N | Y |  |
| *M. fuscata* (captive) | 1309 | 19 | F | N | Y |  |
| *M. fuscata* (captive) | 1350 | 18 | F | Y | Y | 1906(offspring), 1991(offspring) |
| *M. fuscata* (captive) | 1366 | 18 | F | Y | Y | 1796 (offspring),1848(offspring),2065(offspring) |
| *M. fuscata* (captive) | 1394 | 17 | F | N | Y | 2037(offspring), 2067(offspring) |
| *M. fuscata* (captive) | 1458 | 16 | F | N | Y |  |
| *M. fuscata* (captive) | 1466 | 16 | F | N | Y | 1969(offspring) |
| *M. fuscata* (captive) | 1470 | 16 | M | N | Y |  |
| *M. fuscata* (captive) | 1497 | 16 | F | N | Y | 1916(offspring) |
| *M. fuscata* (captive) | 1518 | 15 | F | N | Y | 2091(offspring) |
| *M. fuscata* (captive) | 1605 | 13 | F | Y | Y | 1882(offspring) |
| *M. fuscata* (captive) | 1671 | 12 | F | Y | Y |  |
| *M. fuscata* (captive) | 1683 | 22 | F | N | Y | 1939(offspring) |
| *M. fuscata* (captive) | 1777 | 10 | F | N | Y |  |
| *M. fuscata* (captive) | 1796 | 20 | M | N | Y | 1366 (mother), 1848(sister),2065(sister) |
| *M. fuscata* (captive) | 1848 | 9 | F | Y | Y | 1366(mother), 2065(sister), 1796(brother) |
| *M. fuscata* (captive) | 1882 | 8 | F | N | Y | 1605(offspring) |
| *M. fuscata* (captive) | 1889 | 8 | M | N | Y |  |
| *M. fuscata* (captive) | 1906 | 8 | F | N | Y | 1350(mother), 1991(sister) |
| *M. fuscata* (captive) | 1916 | 7 | F | N | Y | 1497(mother) |
| *M. fuscata* (captive) | 1925 | 7 | F | Y | Y |  |
| *M. fuscata* (captive) | 1939 | 7 | F | N | Y | 1683(mother) |
| *M. fuscata* (captive) | 1954 | 7 | F | N | Y |  |
| *M. fuscata* (captive) | 1969 | 6 | F | N | Y | 1466(mother) |
| *M. fuscata* (captive) | 1991 | 6 | F | N | Y | 1350(mother), 1906(sister) |
| *M. fuscata* (captive) | 2037 | 5 | F | N | Y | 1394(mother), 2067(sister) |
| *M. fuscata* (captive) | 2056 | 5 | M | N | Y |  |
| *M. fuscata* (captive) | 2065 | 4 | F | Y | Y |  |
| *M. fuscata* (captive) | 2067 | 4 | F | N | Y | 1394(mother), 2037(sister) |
| *M. fuscata* (captive) | 2091 | 4 | M | N | Y | 1518(mother) |
| *M. fuscata* (wild) | Beta | 25* | F | Y | Y |  |
| *M. fuscata* (wild) | Blanca | 15* | F | Y | Y |  |
| *M. fuscata* (wild) | Kai | 20* | F | Y | Y | Twin1 (possibly offspring); Twin2 (possibly offspring) |
| *M. fuscata* (wild) | Lucy | 15* | F | Y | Y |  |
| *M. fuscata* (wild) | Mimicha | 7* | F | Y | Y |  |
| *M. fuscata* (wild) | Patrice | 10* | F | Y | Y |  |
| *M. fuscata* (wild) | Tracy | 10* | F | Y | Y |  |
| *M. fuscata* (wild) | Twin1 | 1* | M | Y | Y | Kai (possibly mother) |
| *M. fuscata* (wild) | Twin2 | 1* | M | Y | Y | Kai (possibly mother) |
| *M. fuscata* (wild) | Jeannie | 20* | M | N | Y |  |
| *M. fuscata* (wild) | Ollie | 7* | F | N | Y |  |
| *M. fuscata* (wild) | Penny | 12* | M | N | Y |  |
| *M. fuscata* (wild) | Fiona | NE** | F | N | Y |  |
| *M. fuscata* (wild) | Julia | NE** | F | N | Y |  |
| *M. fuscata* (wild) | Katie | NE** | F | N | Y |  |
| *M. fuscata* (wild) | Larry | NE** | M | N | Y |  |
| *M. fuscata* (wild) | Maude | NE** | F | N | Y |  |
| *M. fuscata* (wild) | Pepper | NE** | F | N | Y |  |
| *M. fuscata* (wild) | Sara | NE** | F | N | Y |  |

*estimated

**not estimated

**Supporting Information Table S4.** Results of likelihood ratio tests for clustering

| **Organism** | **pa*_RAW^a^** | **pb*_RAW^a^** | **pa*_JPEG^a^** | **pb*_JPEG^a^** | **plemur_a*_RAW^b^** | **plemur_b*_RAW^b^** | **plemur_a*_JPEG^b^** | **plemur_b*_JPEG^b^** |
| --- | --- | --- | --- | --- | --- | --- | --- | --- |
| Black lemur + Blue-eyed black lemur | 0.75 | 0.0010 | 0.11 | 0.00049 | NA | NA | NA | NA |
| Japanese macaque | NA | NA | 0.21 | 0.31 | NA | NA | 0.15 | 0 |
| Human | 0.000010 | 0.000010 | 0.000010 | 0.000010 | 0.97 | 0.012 | 0.56 | 0.0030 |

^a^p-value for the likelihood ratio test comparing a mixture of two normal distributions (alternative) to one normal (null), determined empirically by comparison of the observed test statistic to test statistics derived from 100,000 simulations using the maximum likelihood estimate (MLE) parameters from the null

^b^proportion of tests with a lower p-value than that in lemurs, in comparison with 1,000 random samples of equal size in the other species

**Supporting Information Table S5.** Comparing white balance methods using ANOVA

|  | **No white balance** | | **White balance 1 (Weng et al. 2005)** | | **White balance 2 (Adobe® Photoshop®)** | |
| --- | --- | --- | --- | --- | --- | --- |
|  | **F-statistic** | **p-value** | **F-statistic** | **p-value** | **F-statistic** | **p-value** |
| **mean_a** | 3.80 | 2.3 x 10^-3^ | 6.67 | 2.2 x 10^-5^ | 6.36 | 3.5 x 10^-5^ |
| **mean_b** | 7.36 | 8.5 x 10^-6^ | 13.71 | 8.0 x 10^-9^ | 23.29 | 7.7 x 10^-12^ |
| **median_a** | 5.34 | 1.6 x 10^-4^ | 7.55 | 6.5 x 10^-6^ | 7.04 | 1.3 x 10^-5^ |
| **median_b** | 7.23 | 1.0 x 10^-5^ | 14.32 | 4.7 x 10^-9^ | 24.68 | 3.4 x 10^-12^ |
| **mode_a** | 3.35 | 5.1 x 10^-3^ | 4.07 | 1.4 x 10^-3^ | 4.75 | 4.3 x 10^-4^ |
| **mode_b** | 6.69 | 2.2 x 10^-5^ | 12.51 | 2.4 x 10^-8^ | 17.68 | 3.1 x 10^-10^ |
