# Supplementary material for: The Convergent Evolution of Blue Iris Pigmentation in Primates Took Distinct Molecular Paths
Source: Am J Phys Anthropol. 2013 May 2;151(3):398–407. doi: 10.1002/ajpa.22280 (PMC3746105; doi:10.1002/ajpa.22280)
Supplement: Supplementary file 5 [file ajpa0151-0398-SD5.doc]

**Online Supporting Information**

Meyer WK, Zhang S, Hayakawa S, Imai H, and M Przeworski, “The convergent evolution of blue iris pigmentation in primates took distinct molecular paths”

**Section 1. Detailed descriptions of photographic methods**

Photographs of five black lemurs and ten blue-eyed black lemurs were taken by David Haring at the DLC with a Nikon® D700 camera, a Nikon® SB 800 flash mounted on the camera with attached Lumiquest® SoftBox diffuser, and a Micro-Nikkor 60 mm f/2.8 lens, and were provided in both RAW and JPEG format. These photographs were taken indoors, with the majority of light provided by the camera flash, and white balance was custom set at each location using a white card. Additional JPEG photographs of an eleventh blue-eyed black lemur were provided from a previous collection; these photographs were taken with natural lighting in the animal’s enclosure.

Photographs of 42 Japanese macaques housed at the Primate Research Institute (PRI) in Inuyama, Aichi Prefecture, Japan, were taken by PRI staff. These photographs were taken during regular health check-ups with a Canon IXY Digital 930 IS camera with flash. We obtained photographs of 19 free-ranging Japanese macaques in the Choshikei Monkey Park on Shodoshima Island, Kagawa prefecture, Japan, using a Canon PowerShot SD1000 camera and a Canon PowerShot SX 120 IS camera, both with default settings.

Photographs of nine brown spider monkeys were kindly provided by Rebecca Rimbach at the German Primate Center Göttingen; these photographs were obtained using a Nikon Coolpix L120 with default settings.

Photographs of 119 humans of European ancestry (39 RAW, 39 JPEG, 41 both formats), obtained as in Edwards et al. (2012), were kindly provided by Esteban Parra and Melissa Edwards of the University of Toronto Mississauga.

**Section 2.** **White balance**.

The process of stabilizing color to correct for varying background illumination is called white balance adjustment (Huo et al., 2006). A typical white balance algorithm starts with the estimation of illuminance (a measure of the amount of light reaching the object) and then adjusts each dimension of the color space independently (Fairchild, 2005). We compared the effects of two such methods, Weng et al. (2005) and Adobe® Photoshop® automatic white balance adjustment, on the mean, median and mode of iris pixels in CIE L*a*b* color space. To do so, we performed ANOVA on quantile normalized CIE a* and b* data from wild macaques, using a regression model with ß_i_ representing the effect of being a photo of macaque i. Since the ANOVA F statistic represents the ratio of between-macaque variation to between-photo variation, an effective white balance method and summary statistic should produce a large F statistic. In general, both white balance methods produced an improvement over the uncorrected data, and median and mean were more consistent across photographs than mode (Supporting Information Table S5). We therefore decided to use the median after Adobe® Photoshop® automatic white balance adjustment for our analyses using JPEG photographs. We adjusted white balance of RAW files (a minimally processed image file format that was available for some lemur and human photographs) by selecting a small patch of pixels that appeared gray, or “color-neutral,” to the eye as input for the Adobe® Camera Raw for Photoshop® CS4 White Balance tool, and used the median after this correction for further analyses.

**Section 3. Testing consistency of iris selection.**

To ensure that the iris selection method was consistent across users, we compared results from two people (WM and SZ) independently selecting the iris; the resulting a* and b* values were highly correlated (Supporting Information Figure S3).

**Section 4.** **Choosing a color system and summary statistic**.

Quantitative variation in human iris color represented in both HSV and CIEL*a*b* color spaces has been associated with genetic variation, particularly at rs12913832 (Liu et al., 2010; Edwards et al., 2012). In CIE L*a*b*, L* represents brightness, a* represents relative amount of magenta to green, and b* represents relative amount of yellow to blue (Malacara, 2002). In HSV, V represents value, or brightness of color, ranging from 0 to 1. At each value, colors of a constant brightness form a circle, with angle representing hue (H), and radius representing saturation (S), or the difference between the color and gray (Joblove and Greenberg, 1978).

We selected 100 pixels from the same area across multiple photographs of each wild macaque and converted from RGB (the standard space used by digital cameras; Stokes et al., 1996) to HSV with the colorsys module in Python 2.7.1 or MATLAB® R2011a and to CIE L*a*b* using custom code by Mark Ruzon downloaded from http://www.mathworks.com/matlabcentral/fileexchange/24009 on January 25th, 2012. If the color space separates brightness from color well, points from different photographs of the same macaque should overlap on the S-V or a*-b* plane, because lighting condition differences should be absorbed by H or L*. The two spaces are comparable in their ability to represent color independent of lighting (Supporting Information Figure S4). Due to the difficulties inherent in working with circular data such as S-V, we therefore decided to use CIE L*a*b*, focusing on CIE a* and CIE b* to minimize the effects of lighting conditions.

**Section 5. Likelihood ratio test of a normal mixture model against a simple normal model.**

Under the null hypothesis of no genetic loci with strong effects on iris pigmentation, the variation in the phenotype within the a* and b* dimensions of color space should be expected to follow a simple normal distribution. Under the alternative of a locus with strong effect and appreciable dominance (as in humans), the expectation is instead a mixture of two normal distributions, centered at the means of the two genotype classes. To test this particular alternative against the null within each primate group, we fitted a mixture normal model with two components and compared the likelihood to that of a simple normal model using a likelihood ratio test. Because the test statistic does not follow the standard chi-square distribution in this setting (see, e.g., McLachlan, 1987), an empirical distribution of the null (one cluster) model was generated for each species by calculating test statistics of 100,000 datasets simulated using a simple normal model with the maximum likelihood estimate (MLE) parameters from the data under this model. The p-value was then obtained by comparing the observed likelihood ratio test statistic of the mixture model versus the simple normal model to this empirical null distribution.

**Section 6. Sequencing protocol.**

For sequencing, 1.2 μL of purified PCR product and 1.2 μL of 4 μM primer were added to 2 μL BigDye® v3.1 (Applied Biosystems) and 2 μl water, and this reaction mix was cycled 50 times for 5 sec at 96ºC, 10 sec at 50ºC, and 3 min at 60ºC.  Water was added to approximately 25 μl, and reactions were purified by Sephadex® G-50 Superfine (GE Healthcare Bio-Sciences AB) in a 96-well filter plate (Pall).  These were directly placed on the Applied Biosystems 3730XL or 3130 capillary sequencer and run as a standard 2 hour run using POP-7™ polymer (Applied Biosystems).

**Section 7. Neutral expectations of the number of segregating sites.**

The number of segregating sites (S) expected in a region by chance under a simple demographic model and assuming neutrality can be calculated from an estimate of pairwise heterozygosity (π), using the following relation:

(Tajima, 1989).

Although pairwise heterozygosity estimates are not available for the blue-eyed black lemur, black lemur, or Japanese macaque, we estimated the number that would be expected if this heterozygosity were equal to that of a related species. The total number of segregating sites identified in blue-eyed black lemurs (eight, including the SNP identified in Bradley et al. (2009)) is comparable to the six to nine expected by chance, assuming neutrality (using the average nucleotide diversity in the most closely related species for which estimates are available, *Eulemur coronatus*; Perry et al., 2012), and the number identified in black lemurs (two), is slightly less than this expectation. Approximately five segregating sites were expected in captive Japanese macaques based on nucleotide diversity in the most closely related species sequenced (*Macaca mulatta*; Perry et al., 2012), whereas six were identified in this study; in the wild Japanese macaques, two segregating sites were found when roughly two were expected. Thus, assuming the values estimated using diversity data in these related species are accurate, our results suggest that the orthologous region in lemurs or macaques contains roughly the same number of segregating sites as would be expected under neutrality.

**LITERATURE CITED**

Edwards M, Gozdzik A, Ross K, Miles J, Parra EJ. 2012. Technical note: Quantitative measures of iris color using high resolution photographs. Am J Phys Anthropol 147(1):141-149.

Fairchild MD. 2005. Color appearance models. 2nd ed. Chichester, West Sussex, England ;Hoboken, NJ: J. Wiley.

Huo J, Chang Y, Wang J, Wei X. 2006. Robust automatic white balance algorithm using gray color points in images. Consumer Electronics, IEEE Transactions on 52(2):541-546.

Joblove GH, Greenberg D. 1978. Color spaces for computer graphics. SIGGRAPH Comput.Graph. 12(3):20-25.

Malacara D. 2002. Color vision and colorimetry : Theory and applications. Bellingham, WA: SPIE Press.

McLachlan GJ. 1987. On bootstrapping the likelihood ratio test stastistic for the number of components in a normal mixture. Journal of the Royal Statistical Society.Series C (Applied Statistics) 36(3):318-324.

Perry GH, Melsted P, Marioni JC, Wang Y, Bainer R, Pickrell JK, Michelini K, Zehr S, Yoder AD, Stephens M, Pritchard JK, Gilad Y. 2012. Comparative RNA sequencing reveals substantial genetic variation in endangered primates. Genome Res 22(4):602-610.

Stokes M, Anderson M, Chandrasekar S, Motta R. 1996. A standard default color space for the internet - sRGB. http://www.w3.org/Graphics/Color/sRGB ed.

Tajima F. 1989. Statistical method for testing the neutral mutation hypothesis by DNA polymorphism. Genetics 123(3):585-595.

Weng C, Chen H, Fuh C. 2005. A novel automatic white balance method for digital still cameras. In: Anonymous IEEE International Symposium on Circuits and Systems, 2005. ISCAS 2005. p 3801-3804 Vol. 4.
